# IESSP: Information Extraction-Based Sparse Stripe Pruning Method for Deep Neural Networks

**DOI:** 10.3390/s25072261

**Published:** 2025-04-03

**Authors:** Jingjing Liu, Lingjin Huang, Manlong Feng, Aiying Guo, Luqiao Yin, Jianhua Zhang

**Affiliations:** Shanghai Key Laboratory of Chips and Systems for Intelligent Connected Vehicle, School of Microelectronics, Shanghai University, Shanghai 200444, China; jjliu@shu.edu.cn (J.L.); 22723714@shu.edu.cn (L.H.); emxlight22@shu.edu.cn (M.F.); gayshh@shu.edu.cn (A.G.); lqyin@shu.edu.cn (L.Y.)

**Keywords:** network pruning, information extraction-based sparse stripe pruning (IESSP), information extraction module (IEM), adaptive optimization

## Abstract

Network pruning is a deep learning model compression technique aimed at reducing model storage requirements and decreasing computational resource consumption. However, mainstream pruning techniques often encounter challenges such as limited precision in feature selection and a diminished feature extraction capability. To address these issues, we propose an information extraction-based sparse stripe pruning (IESSP) method. This method introduces an information extraction module (IEM), which enhances stripe selection through a mask-based mechanism, promoting inter-layer interactions and directing the network’s focus toward key features. In addition, we design a novel loss function that links output loss to stripe selection, enabling an effective balance between accuracy and efficiency. This loss function also supports the adaptive optimization of stripe sparsity during training. Experimental results on benchmark datasets demonstrate that the proposed method outperforms existing techniques. Specifically, when applied to prune the VGG-16 model on the CIFAR-10 dataset, the proposed method achieves a 0.29% improvement in accuracy while reducing FLOPs by 75.88% compared to the baseline.

## 1. Introduction

In recent years, deep neural networks (DNNs) have achieved remarkable success across various computer vision applications, such as image classification [1], object detection [2], video analysis [3], and semantic segmentation [4]. The effectiveness of these networks is attributed to depth and width, resulting in complex computations and a substantial demand for storage units, which makes the models unsuitable for deployment on resource-constrained edge devices [5]. Consequently, many researchers have proposed model compression methods, including network pruning [6], knowledge distillation [7], low-rank decomposition [8], and quantization [9]. Network pruning has become increasingly popular in real-time neural network deployment at the network edge because it maintains model performance without changing the original architecture and processes effectively with other compression techniques.

As a critical technique for selecting acceleration modes, network pruning is typically categorized into structured general-purpose pruning and unstructured custom pruning. Unstructured pruning operates by removing individual weights to reduce the number of parameters, aiming to create irregular sparse networks while minimizing accuracy loss [10,11]. However, irregular sparsity can disrupt memory access patterns, resulting in decreased efficiency during inference or training on hardware accelerators. Therefore, specialized hardware is required to achieve effective acceleration for unstructured pruning. In contrast, structured pruning targets specific components, allowing for acceleration on general-purpose platforms without the specialized hardware [12]. For instance, filter pruning reduces model size by removing entire channels within network layers, but the loss of intrinsic components limits the performance improvement of the network [13,14]. More recently, stripe pruning, which is a finer-grained structured pruning method, has gained significant attention [15,16]. Stripe pruning avoids directly removing intrinsic components of the network and offers a more flexible pruning method through sparse selection compared to filter pruning. However, most existing stripe pruning methods concentrate on optimizing filter shapes and parameter selection but do not fully utilize the intra-filter information. Additionally, the stripe pruning methods typically impose global sparsity constraints during pruning, neglecting the adaptability between subnetworks and specific tasks, which will lead to diminished network performance.

The pruning criterion is another key factor for the effective implementation of network pruning [17]. Commonly used pruning criteria include methods based on magnitude, norms, significance, and loss functions. Magnitude-based pruning [18] assumes that parameters with smaller absolute values exert less influence on the network output, and the norm-based methods [19] define specific rules, such as sparse $L_{1}$ or $L_{2}$ regularization [20,21] to evaluate the importance of parameters. Significance or sensitivity pruning [22] ranks weights based on the respective contribution to the network, while loss-based methods [23] assess the importance of weights by comparing changes in network loss with and without the weight. These methods offer different perspectives on determining which parameters or structures to prune, depending on the relative importance to the overall network performance. While single-criterion pruning is straightforward, the lack of flexibility still limits the complex information interactions within the neural network. Moreover, pruning criteria require significant parameter differences between important and unimportant filters. However, methods solely based on norm regularization or loss functions often fail to effectively drive the elements of the parameter matrix toward zero, resulting in suboptimal pruning performance.

**Figure 1:**
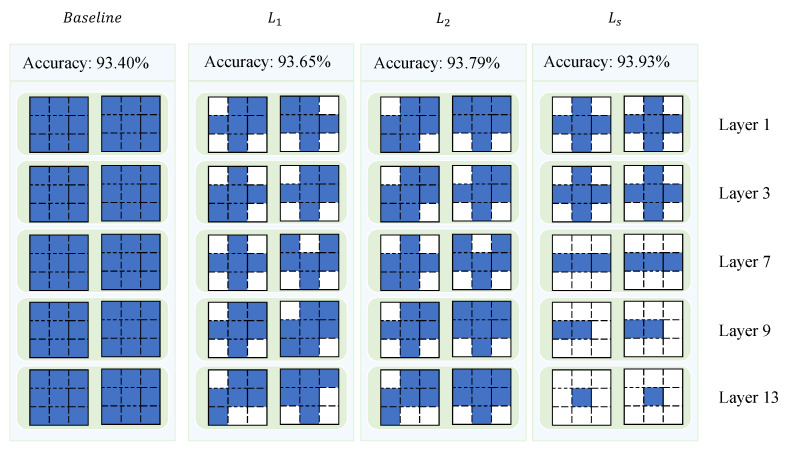
Parts of each layer with different regularization.

Motivated by the works above, this paper introduces an information extraction-based sparse stripe pruning method (IESSP). IESSP utilizes internal network feature information to guide sparse feature extraction, integrating it with stripe selection to enhance both precision and efficiency in pruning. At a finer granularity, filters extract information using diverse shape matrices, which are then incorporated into the decision-making process of stripe pruning, improving the overall network performance. To address potential instability from excessive sparsity, we propose an adaptive sparsity training strategy. In contrast to the sole use of $L_{1}$ and $L_{2}$ regularization, this strategy incorporates feature information with task-specific $L_{S}$ sparsity regularization, embedding it into the objective function for optimized sparsity selection. The difference between the layers of the proposed method and existing methods is depicted in Figure 1. Through this approach, IESSP captures the relationship between training loss and sparsity configuration, enabling adaptive optimization. Experimental results on benchmark datasets demonstrate that IESSP outperforms existing techniques.

The major contributions of this paper are as follows:

- Novel pruning method. We propose an information extraction-based sparse stripe pruning method (IESSP) that adaptively adjusts sparsity during training. By leveraging sparsity regularization, it guides filter stripe selection through information extraction. The method incorporates an information extraction module (IEM) to further enhance stripe selection by improving layer interactions and focusing the network on key patterns.
- Novel loss function. We propose a novel loss function that introduces adaptive sparsity in neural networks by dynamically adjusting the sparsity of the filter skeleton through regularization. This approach strikes a balance between performance and compactness, enhancing feature selection efficiency and reducing redundancy while preserving model performance.
- Extensive experiments. We conduct experiments across multiple datasets and architectures, comparing our method with state-of-the-art techniques to demonstrate its effectiveness.

The paper is organized as follows: Section 2 reviews network pruning techniques and sparsity regularization methods. Section 3 introduces the IESSP method and its efficient implementation using optimization algorithms. Section 4 demonstrates the application of IESSP to various neural networks for image classification tasks on different datasets. Section 5 concludes with a comprehensive summary.

## 2. Related Works

This section first provides a brief overview of the development of traditional pruning methods, followed by an introduction to the application of regularization-based sparse training in network pruning.

### 2.1. Network Pruning

Pruning is a model compression technique that reduces computational load and memory requirements by removing redundant parameters from neural networks. The primary goal of pruning is to enhance model efficiency while maintaining original predictive accuracy as much as possible. The most basic pruning method is filter and convolution kernel level pruning [24,25]. By pruning the entire filter or convolution kernel, the model size and computational complexity can be significantly reduced, resulting in an improvement in inference speed. However, due to excessive pruning, this pruning method usually leads to a decrease in model performance. It is difficult to control the granularity of pruning. Therefore, neuron and parameter level pruning is proposed, which iteratively removes the part with the smallest contribution by evaluating the contribution of neurons or parameters to the final loss [26,27,28]. This strategy can control the pruning granularity more effectively, further reducing the model size and computational complexity. However, this pruning method requires evaluation and iteration processes, resulting in increased training time. Based on the above pruning methods, channel pruning optimizes the model structure and performance by selectively pruning the channels in the network [13,29]. But the channel selection process is relatively complex, which has led to the emergence of structured pruning [24,30,31]. Structured pruning methods rely on a complete pruning process to produce a more effective final model. By modifying the training strategy, this strategy occasionally increases the complexity of model training. Therefore, the pruning methods for specific tasks have been proposed [32,33]. When facing specific tasks, these methods improve the pruning performance by reducing the generality and flexibility of the models. In summary, recent developments in pruning have shown a clear trend toward combining structured pruning methods with adaptive, task-specific strategies. However, challenges remain in balancing pruning-induced sparsity with task performance, especially when dealing with fine-grained structures or complex architectures.

### 2.2. Sparsity Regularization Based Methods

Sparsity regularization-based methods aim to extract sparsity from dense networks by reducing the number of weights, connections, filters, or neurons, thereby lowering memory usage and computational complexity. Tensor completion and sparse induction methods use tensor trace norms to handle missing values and introduce sparsity regularization to promote sparsity in network layers [34,35]. However, these methods occasionally result in information loss or low reconstruction accuracy. On this basis, the number of neurons and structured sparse optimization methods significantly optimize the network structure and reduce computational complexity by automatically determining the number of neurons in each layer and adopting structured sparsity [36,37]. But the process of automatically determining the number of neurons is relatively complex, and it is difficult to accurately find the optimal network structure. Therefore, the convolutional kernel sparse pruning strategy has been proposed, which involves aggressive pruning of convolutional kernels and requires significant time to balance the trade-off between pruning intensity and model performance [38]. To solve this issue, sparse connection learning and two-stage pruning strategies are proposed to provide support for building efficient and streamlined neural network models, offering more reliable solutions for practical applications [39,40,41]. The method of selective sparsity regularization has also garnered attention [42]. Although structured sparsity regularization has shown potential in network pruning, task-specific sparsity adjustment strategies for complex network architectures require further exploration.

### 2.3. Stripe Pruning

Stripe pruning, as a fine-grained network pruning method, achieves network compression by precisely identifying and removing low-contribution stripes within filters, in contrast to traditional filter pruning methods. Meng et al. [43] proposed a relatively simple yet efficient stripe pruning method that uses a sparse regularization-based mask to identify important stripes in the filters, thereby enabling network compression. Specifically, the core idea of stripe pruning is to control the contribution of each stripe in the convolutional kernel through a sparse mask $S_{l}^{n,i,j}$, where *l* denotes the index of the convolutional layer, *n* denotes the sample index, i,j are the spatial indices of the convolutional kernel, and $S_{l}^{n,i,j}$ is the mask value for each stripe, determining whether the stripe is retained.

In particular, the calculation of stripe convolution can be expressed as follows:

(1) $$X_{l+1}^{n,h,w}=\sum_{c=1}^{C}\sum_{i=1}^{K}\sum_{j=1}^{K}S_{l}^{n,i,j}\cdot W_{l}^{n,c,i,j}\cdot X_{l}^{n,h+i-\frac{K}{2},w+j-\frac{K}{2}},$$

where $S_{l}^{n,i,j}$ is the importance mask for the stripes in the filter, $W_{l}^{n,c,i,j}$ represents the filter weights, and $X_{l}^{n,h,w}$ denotes the input feature map. Stripe pruning introduces the mask $S_{l}^{n,i,j}$ into the convolution operation, retaining only the stripes that contribute significantly to the classification task and removing low-contribution stripes. This results in reduced network parameters and improved computational efficiency. During inference, the pruned network performs the convolution operation with each retained stripe independently, and the feature maps produced by each stripe are then summed together. This process can be mathematically described as:

(2) $$X_{l+1}^{n,h,w}=\sum_{i=1}^{K}\sum_{j=1}^{K}\left(\sum_{c=1}^{C}W_{l}^{n,c,i,j}\cdot X_{l}^{n,h+i-\frac{K}{2},w+j-\frac{K}{2}}\right),$$

where the convolution is performed for each stripe individually, and the summation of the resulting feature maps produces the final output.

Liu et al. [44,45] focuses on designing specialized convolutional kernel shapes for stripe pruning, but they do not account for the correlation between different layers. Therefore, recent research has begun to consider the balance of stripes across different layers and adaptive stripes pruning to further improve network compression efficiency [46]. However, despite the ability of stripe pruning methods to offer more precise pruning control, they still face challenges in effectively transmitting and sharing stripe information between layers while maintaining network performance.

## 3. Methodology

**Figure 2:**
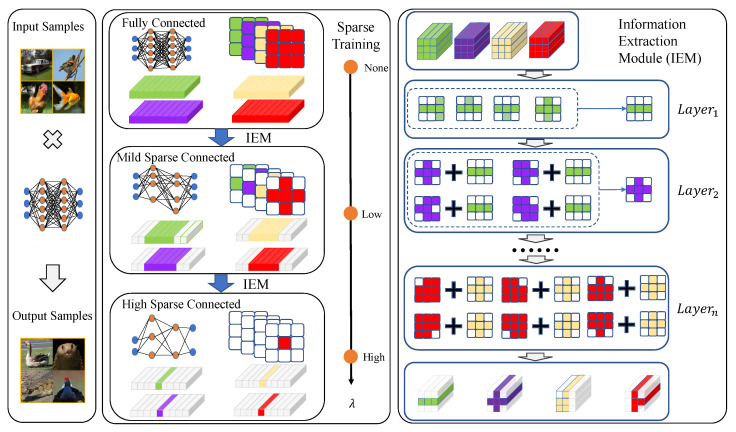
The schematic diagram of sparse training and information extraction module in IESSP, where cubes in different colors represent the filters in different layers, with depth equal to the number of input channels *C*, and width and height corresponding to the width and height of the convolutional kernel *K*. The figure demonstrates the effect of using different binary masks during the inference process.

This section primarily focuses on the proposed sparse stripe pruning method. In Section 3.1, we present the overall framework of the approach, along with a regularization method designed for sparse feature extraction. The proposed sparse training and IEM are illustrated in Figure 2. In Section 3.2, a $L_{S}$ sparse training strategy is introduced to optimize the balance between network sparsity and feature extraction capabilities.

### 3.1. The IESSP Framework Architecture

This study introduces a novel approach that integrates feature information extraction and sparsity regularization. By incorporating a self-feedback mechanism, the method dynamically balances performance with sparsity, enabling the network to efficiently learn both compact and expressive representations. Central to this framework is the sparsity regularization term, G(S), defined as:

(3) $$G\left(S\right)=\sum_{l}^{L}{∥S_{l}∥}_{p},$$

where $S_{l}=\left[S_{l}^{1,1,1},S_{l}^{1,1,2},\cdots ,S_{l}^{N,K,K}\right]$ is the vector of the convolution kernel masks for layer *l*.

(4) $${∥S_{l}∥}_{p}={\left(\sum_{n=1}^{N}\sum_{i=1}^{K}\sum_{j=1}^{K}{\left|S_{l}^{n,i,j}\right|}^{p}\right)}^{1/p},$$

where *K* represents the kernel size, and *N* denotes the number of filters. ${∥\cdot ∥}_{p}$ represents the $\ell_{p}$-norm used to quantify the importance of filter stripes. By penalizing less significant elements, the regularization term induces selective sparsity, allowing the network to prioritize critical features during training.

To enhance flexibility, a mixed-norm formulation is introduced, defined as:

(5) $$G\left(S\right)=\sum_{l}^{L}\left({∥S_{l}∥}_{1}+{∥S_{l}∥}_{2}\right),$$

which eliminates ineffective stripes while preserving the model’s ability to capture essential features and maintain its predictive performance. However, sparsity regularization alone is insufficient to fully exploit feature interdependencies across layers.

To address this issue, we propose a novel pruning approach via a mask-based mechanism applied along the direction of the convolutional kernel shape (stripe direction), implemented in the information extraction module (IEM). Specifically, the IEM module utilizes the average mask shape from the previous layer to impose a special constraint on the filter shape of the subsequent layer. This approach ensures hierarchical balance across layers, achieving a more efficient pruning mechanism. Let us define the transformation of feature masks between consecutive layers. The feature mask of the current layer is used to modulate the feature mask of the next layer, $S_{l+1}^{i}$, by applying the average convolution kernel mask, $\frac{1}{N}\sum_{n=1}^{N}M_{l}^{n}$, derived from the previous layer. The equation for this interaction can be described as:

(6) $$S_{l+1}^{i}=\frac{1}{N}\sum_{n=1}^{N}M_{l}^{n}+S_{\mathrm{raw},l+1}^{i},$$

where $M_{l}^{n}$ represents the mask of the *n*-th filter in the previous layer, and *N* is the total number of filters in that layer, $S_{\mathrm{raw},l+1}^{i}$ is the initial mask of the next layer. The formula for calculating the average convolution kernel mask $\frac{1}{N}\sum_{n=1}^{N}M_{l}^{n}$ in Equation (6) assumes that the kernel size $K_{l}$ is the same across all layers.

During the training process, the convolution kernel masks are continuously updated. During inference, stripes are pruned in subsequent convolution operations when the mask values fall below a predefined threshold T. This mechanism allows pruning to dynamically adjust based on the importance of each stripe within the network. To ensure consistency between pruning and shape optimization, we integrate the stripe statistics of all layers into the loss function as a constraint. This ensures that pruning aligns with inter-layer shape optimization, preventing pruning from disrupting the overall architecture’s performance.

### 3.2. Loss Function Design for Adaptive Sparsity Optimization

In contrast to traditional methods that rely on pre-training and over-parameterized networks, the proposed method directly learns from the original network. To emphasize the importance of individual stripes within each filter, it progressively constructs a more compact structure by identifying redundant components and incorporating convolution kernel masks. However, the introduction of these convolution kernel masks may affect network performance. To achieve a more compact and efficient structure while controlling the sparsity of the convolution kernel masks, we introduce regularization constraints into the optimization process. These constraints ensure that redundant features are reduced, while the performance of the network is preserved. The total training loss function is defined as follows:

(7) $$L_{total}=L_{ce}(f\left(\left(X,\left(W,S\right)\right),Z\right)+H\left(C_{1}-C_{2}\right)\cdot \lambda L_{S},$$

where $W=\left[W_{1},W_{2},\cdots ,W_{l}\right]\in R^{N\times C\times K\times K}$ denotes the parameters of the *L* convolutional layers in the network. The cross-entropy loss, $L_{ce}\left(f\left(X,\left(W,S\right)\right)\right)$, represents the difference between the predicted output and the true label, (W,S) denotes that the mask is updated with the network, Z, where Z=N(X,W,S) is the output vector of the target network, and λ denotes the weight coefficient. Additionally, H(·) refers to the Heaviside step function, $C_{1}$ represents the current loss value, and $C_{2}$ represents the average loss value from the previous epoch.

To further enhance sparsity and optimize network performance, we refine the control over the convolution kernel masks by introducing additional regularization terms. These terms impose constraints on the sparsity of the convolution kernel masks, progressively forming a more compact structure. The optimization objective is defined as:

(8) $$\begin{array}{c} L_{S}=\sum_{l}^{L}\sum_{n}^{N}\sum_{i}^{K}\sum_{j}^{K}M_{l}^{n,i,j}+\frac{\sum_{l}^{L}\left({∥S_{l}∥}_{1}+{∥S_{l}∥}_{2}\right)}{2}, \end{array}$$

where $\sum_{l}^{L}\sum_{n}^{N}\sum_{i}^{K}\sum_{j}^{K}M_{l}^{n,i,j}$ represents the stripe statistics of all layers, which serve as a constraint to ensure consistency between pruning and inter-layer shape optimization. The second part of the equation, ${∥S_{l}∥}_{1}+{∥S_{l}∥}_{2}$, represents the mixed regularization term applied to the masks. Here, ${∥S_{l}∥}_{1}$ encourages sparsity in the convolution kernel masks, promoting the pruning of less important features, while ${∥S_{l}∥}_{2}$ helps to smooth the mask values, ensuring that the model does not become excessively sparse in an unstructured manner. The combination of both regularization terms helps balance the pruning process by controlling the sparsity and smoothness of the kernel masks, thereby optimizing feature retention during pruning.

In this context, the weight coefficient λ plays a pivotal role in regulating the sparsity penalty applied to stripe significance. Networks that achieve an optimal level of sparsity tend to minimize loss more efficiently, demonstrating that compact network architectures can enhance computational efficiency across various tasks. However, when sparse networks underperform on specific tasks, it becomes crucial to moderate the sparsity process to balance task performance with network sparsity. The optimization process is further complicated by the interplay of multiple constraints, such as weight decay and the convolution kernel mask sparsity. This complexity often leads global sparsity techniques to converge more slowly, ultimately degrading performance. To address these challenges, the proposed method shifts away from a global sparsity learning approach for the convolution kernel masks and adopts an independent sparsity learning strategy. This targeted approach offers more effective optimization by addressing sparsity at a finer level. Unlike traditional methods that depend on empirically selected hyperparameters, the proposed approach dynamically adjusts the sparsity strength based on the evolving loss state during network training. This adaptive mechanism eliminates the need for manual hyperparameter tuning, enhancing both the flexibility and robustness of the training process. By balancing sparsity during training, the strategy ensures that the network achieves an optimal equilibrium between performance and sparsity at various stages. As training progresses, the sparsity level is adjusted based on internal feedback, enabling the network to achieve maximum compression while preserving performance. This dynamic adaptation not only improves computational efficiency but also reduces the risk of overfitting.

To further promote sparsity in the mask, the proposed method introduces an additional term into the loss function, specifically designed to encourage a more distinct sparse configuration. This penalty term imposes stricter constraints on the mask values, effectively promoting the suppression of less significant stripes. As a result, the model refines its feature selection process, producing a more compact and efficient representation of the underlying data. The penalty term not only helps eliminate redundant information but also maintains a balance between sparsity and performance, ensuring that critical features are preserved while minimizing redundancy. By guiding the optimization towards masks with minimal non-zero elements, this approach fosters the creation of a more sparse and interpretable model structure. Importantly, the penalty term ensures that the model maintains satisfactory performance without compromising its ability to capture essential patterns from the input data, thereby guaranteeing both efficiency and effectiveness. Furthermore, the proposed method integrates feedback during training, refining the calculation of cross-entropy and enhancing information exchange between layers. This improves the network’s robustness while ensuring the selection and representation of critical features, achieving a better balance between sparsity and performance. By combining feature integration with sparse regularization, the method not only improves model performance but also optimizes efficiency in practical applications.

## 4. Experiment

This section presents comprehensive experimental results to analyze the proposed IESSP method. Network pruning experiments are conducted on the CIFAR-10 dataset [47], utilizing the single-branch network VGG-16 [48] and multi-branch networks, including ResNet20, ResNet32, and ResNet56 from the ResNet family [49]. Furthermore, compact models such as ResNet18 and ResNet34 were trained on the large-scale ILSVRC-2012 dataset [50]. Table 1 provides comprehensive descriptions of the datasets and network architectures, along with the rationale for their selection. This ensures a fair and meaningful comparison with state-of-the-art methods, including PFEC [24], FPGM [30], NISP [51], GAL [52], HRank [31], EACP [53], SOKS [45], and SWP [43].

### 4.1. Datasets and Experimental Setup

For CIFAR-10, standard data augmentation techniques, including random cropping and horizontal flipping, were applied to enhance generalization. Training was conducted for 160 epochs using the stochastic gradient descent (SGD) optimization algorithm, with a momentum of 0.9, a weight decay of $10^{-4}$, and a batch size of 64. The initial learning rate was set to 0.1 and reduced by a factor of 0.1 at the 80th and 120th epochs. To control the pruning process, the pruning threshold T was fixed at 0.05, according to the reference [43]. The sparsity coefficient λ was set to $10^{-5}$.

For ILSVRC-2012, standard preprocessing techniques, such as random cropping and flipping, were applied during training, while a center crop of size 224×224 was used for testing. The training spanned 90 epochs, with the initial learning rate starting at 0.1 and being reduced by a factor of 0.1 every 30 epochs. The same SGD optimization parameters—momentum of 0.9 and weight decay of $10^{-4}$—were utilized. Similar to CIFAR-10, the pruning threshold T and sparsity coefficient λ were kept consistent at 0.05 and $10^{-5}$, respectively.

All models were implemented in PyTorch (version 2.3.0) and trained on NVIDIA RTX 4090 GPUs, with CUDA version 12.4. The GPUs were manufactured by NVIDIA and sourced from the USA. The smaller scale and lower resolution of CIFAR-10 make it an accessible but nontrivial benchmark for exploring the proposed pruning method, particularly for compact models. Conversely, the large scale and complexity of ILSVRC-2012 allow for a more comprehensive evaluation of scalability, feature extraction, and generalization capabilities across diverse categories. Key evaluation metrics, such as the number of model parameters, floating-point operations (FLOPs), and classification accuracy, were employed to assess model compression and performance. Consistent with existing methodologies, only top 1 accuracy was reported for CIFAR-10, while both top 1 and top 5 accuracies were included for ILSVRC-2012.

#### Performance Evaluation

**Figure 3:**
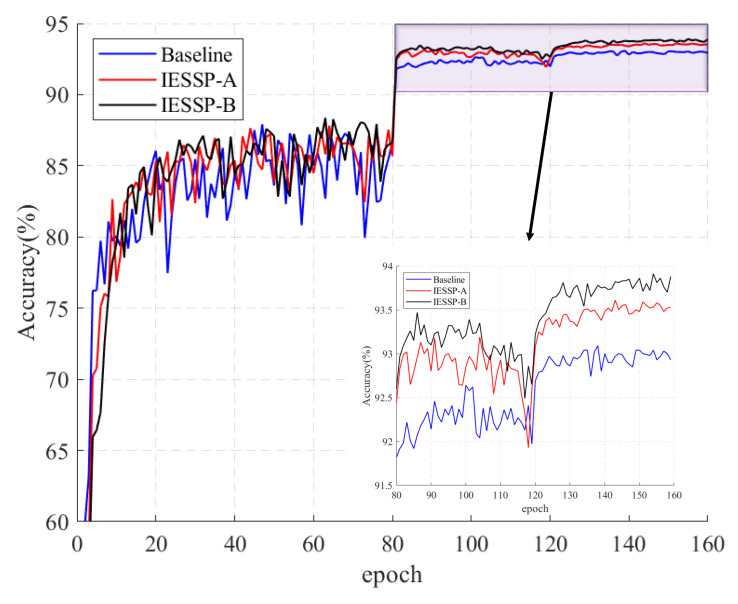
Accuracy comparison of IESSP with different module additions and baseline model for compressing VGG-16 on CIFAR-10.

Table 2 presents the pruning results for four prominent network architectures on the CIFAR-10 dataset. The proposed IESSP method effectively reduces both FLOPs and parameter counts by over 50% while maintaining or exceeding baseline accuracy. For example, in the case of VGG-16, the IESSP method achieves a 75.88% reduction in FLOPs and a 94.6% decrease in the number of parameters compared to the baseline model. Notably, the accuracy improves to 93.69%, marking a 0.29% increase over the baseline. Figure 3 presents a comparison of the accuracy changes between the IESSP method and the baseline model. These results demonstrate IESSP’s effectiveness and its potential for deploying VGG networks on mobile and edge devices.

The IESSP is also applied to three ResNet architectures: ResNet-56, ResNet-32, and ResNet-20. On CIFAR-10, using IESSP, ResNet-56 achieved a 0.59% increase in accuracy, reaching 93.65%, while reducing FLOPs and parameters by 61.3% and 58.8%, respectively. For ResNet-32, the pruned network exhibited a slight accuracy improvement of 0.02%, achieving 93.84%, alongside a 60.5% reduction in FLOPs and a 54.3% reduction in parameters. However, ResNet-20 experienced a slight decrease in accuracy by 0.78%, resulting in a final accuracy of 91.27%, despite significant reductions of 60.6% in FLOPs and 51.9% in parameters. These findings underscore the efficacy of IESSP in pruning multi-branch network structures like ResNets and highlight its ability to consistently enhance the performance of lightweight ResNets across different depths.

To further validate the effectiveness of IESSP, the proposed method is applied to the large-scale ILSVRC-2012 dataset, which contains 1000 categories. Experiments were conducted on two compact ResNet architectures, ResNet18 and ResNet34, with results summarized in Table 3. The compressed network structures exhibited slightly lower accuracy than the original models, which can be attributed to two key factors. First, ResNet architectures are inherently compact, with fewer redundant parameters. Second, the ILSVRC-2012 dataset’s complexity, with its 1000 categories, makes network compression particularly challenging. Despite these challenges, IESSP demonstrates its effectiveness in network pruning. With a pruning threshold of 0.05 applied to ResNet18, the method achieves a 44% reduction in FLOPs and a 36.2% reduction in parameters, while the top 1 and top 5 accuracies decrease by 2.27% and 1.71%, respectively, compared to the baseline.

### 4.2. Comparison with Other Methods

In this section, to validate the effectiveness of the proposed method, IESSP is compared with other state-of-the-art compression methods on the CIFAR-10 and ILSVRC-2012 datasets. The comparative results are presented in Table 4 and Table 5.

Table 4 presents the results of applying IESSP to the VGG-16 model for the CIFAR-10 classification task and compares it with other state-of-the-art techniques. For methods like PFEC, which perform filter pruning using a regularization sparsity ratio factor, IESSP outperforms with an accuracy of 93.69% while compressing the model by a factor of 20. For methods like FPGM, which use geometric median-based importance determination, IESSP also excels in accuracy and compression rate, 6.7 times smaller than the compressed FPGM model while achieving improved accuracy. This is because IESSP targets internal filter redundancy at a finer granularity than filters, achieving good pruning results while maintaining task accuracy. Compared to stripe-wise pruning methods like SWP, IESSP enhances model performance in terms of accuracy and demonstrates further potential in model compression. In the case of kernel optimization methods like ASK, IESSP achieves a six-fold higher compression ratio while maintaining higher accuracy. This is because kernel optimization methods are designed for specific kernel shapes, neglecting the complex receptive fields required by the network. IESSP, on the other hand, does not restrict filter shapes, allowing the learning matrix to choose freely and preserving more receptive fields, resulting in improved performance. For methods like SOKS, which involve designing kernel shapes in groups, IESSP achieves a higher compression ratio than SOKS and performs better in accuracy, reaching 94.16%. In summary, IESSP retains high network compression ratios and maximizes network performance thanks to the richness of the learning matrix. Experimental results demonstrate that the IESSP method proposed in this paper shows superior performance in lightweight neural networks with simple structures and provides a method for deploying VGGNet structures on mobile devices.

Table 4 demonstrates the IESSP method’s impact on ResNet-20 and compares it with other state-of-the-art models. ResNet-20 is a lightweight network, and compressing this network can be challenging. Compared to the MIL method, IESSP compresses ResNet-20 by 40% of the original model while maintaining an accuracy of 91.27%, only 0.16% lower than MIL. Compared to pruning models using the geometric median method, FPGM, IESSP achieves a higher compression rate, compressing FLOPs by an additional 8.3 M and achieving 0.44% higher accuracy than FPGM. For the ASK method, which prunes models using specified kernel shapes, IESSP outperforms in compression rate, compressing by an additional 0.02 M FLOPs, though with 0.5% lower accuracy than ASK. In the case of SOKS, a method that uses groups to specify shapes, IESSP compresses more parameters than SOKS and exhibits better model performance, with an accuracy 0.5% higher than SOKS.

Table 4 demonstrates the impact of the IESSP method on ResNet-32 and compares it with other state-of-the-art pruning techniques. Compared to the MIL method, IESSP achieves a higher compression rate, reducing the model size while maintaining an accuracy of 92.84%, which is 2.12% higher than MIL. When compared to the SFP method, both of which use a soft filter pruning strategy, IESSP improves accuracy by 2.76%. FPGM, another established pruning method, results in a lower compression rate than IESSP and achieves an accuracy that is 0.53% lower. ASK, which prunes based on specified kernel shapes, is another competitive method, but IESSP achieves better accuracy, with an improvement of 0.65%, though its compression rate is slightly lower than ASK. Compared to the SOKS method, IESSP outperforms it in terms of accuracy, demonstrating the effectiveness of IESSP in balancing model size and predictive performance.

Table 4 showcases the impact of the IESSP method on ResNet-56 and compares it with other state-of-the-art models. ResNet-56 is a lightweight network, and compressing this network can be challenging. Compared to the PFEC method, IESSP compresses ResNet-56 by 61.3% of the original model while achieving an accuracy of 93.65%, surpassing MIL’s classification accuracy by 0.59%. Compared to the NISP method for model pruning, IESSP achieves a higher compression rate, compressing FLOPs by an additional 0.14 M and achieving 0.66% higher accuracy than NISP. Compared to the GAL method for model pruning, IESSP achieves a slightly lower compression rate by 0.04 M FLOPs but outperforms GAL in accuracy, achieving a 0.57% higher accuracy. Regarding the SWP method, which prunes models using filter skeleton search, IESSP excels in classification accuracy, compressing by 0.16 M FLOPs less than ASK but achieving 0.67% higher classification accuracy. For the ASK method, which prunes models using specified kernel shapes, IESSP outperforms in compression rate, compressing by 0.13M FLOPs more than ASK, and achieves 0.63% higher accuracy than ASK. In the case of SOKS, a method that uses groups to specify shapes, IESSP compresses 0.04 M fewer parameters than SOKS and exhibits better model performance with 0.57% higher accuracy than SOKS.

Table 5 presents the performance of the IESSP method on ResNet-18 and ResNet-34, comparing it with other state-of-the-art model compression techniques. ResNet-18, being a relatively lightweight network, faces challenges in maintaining high accuracy while compressing. Compared to the baseline, IESSP reduces the number of parameters in ResNet-18 by 48.25% and FLOPs by 56.59%, with only a 1.09% drop in top 1 accuracy, achieving 69.33%. Compared to the MIL method, IESSP not only achieves a significant improvement in compression rate but also retains high performance, with a 0.56% increase in top 1 accuracy. When compared to the SFP and FPGM methods, IESSP shows similar compression rates but higher accuracy, particularly surpassing FPGM by 0.99% in top 1 accuracy. Additionally, in comparison with the SOKS method, while IESSP compresses parameters 0.22M less than SOKS, its top 1 accuracy improves by 0.57%, demonstrating its advantage in balancing compression and accuracy. For ResNet-34, IESSP also performs impressively. Compared to the baseline, IESSP achieves a significant advantage in compression, reducing the number of parameters by 53.90% and FLOPs by 58.96%, while maintaining a top 1 accuracy of 73.18%, only a 0.83% drop from the baseline’s 74.01%. Compared to the PFEC method, IESSP improves top 1 accuracy by 1.01%, showing excellent performance despite a slightly lower compression rate. Compared to the ASK method, IESSP not only excels in compression rate, reducing parameters by 42.98% and FLOPs by 42.66%, but also improves top 1 accuracy by 0.57%. In comparison with the NISP method, IESSP compresses FLOPs further while maintaining a high top 1 accuracy of 73.18%, demonstrating its robust compression capability in large-scale networks.

The comparative results indicate that the IESSP method can significantly reduce network redundancy while maintaining network performance, making it applicable to both large models and lightweight models, as well as multi-branch networks.

### 4.3. Ablation Studies

This section presents a comprehensive analysis of the impact of various hyperparameters on model training and the resulting pruning outcomes. Through ablation experiments, this paper explores the impact of adjusting specific hyperparameters on the training process and pruning strategy. By examining these factors in detail, the experiments aim to provide insights into optimizing the balance between model accuracy and computational efficiency.

#### 4.3.1. Impact of Different Threshold

The pruning threshold T is a critical factor in selecting stripe features and plays a key role in regulating sparsity effectively [43]. An excessively high threshold may lead the model to misclassify important features as redundant, thereby diminishing its representational capacity. Conversely, a threshold that is too low may fail to adequately eliminate redundant structures, limiting the effectiveness of pruning.

In the experiments conducted on the CIFAR-10 dataset, the VGG-16 model was used as the baseline for image classification, systematically exploring the impact of varying pruning thresholds. As shown in Table 6, the IESSP method yielded different pruning outcomes at different threshold levels. Notably, with a threshold of 0.03, the model efficiently identifies and removes redundant components, achieving an accuracy of 94.16%, while reducing the parameter count by a factor of five and cutting FLOPs by 50%.

However, as the threshold increases, excessive sparsity begins to degrade classification accuracy, even though network compression continues to improve. These results highlight the delicate balance required in selecting an optimal pruning threshold for model performance. While aggressive pruning can significantly reduce the model size and computational demand, there is a critical threshold beyond which further compression results in diminishing returns on accuracy. This underscores the importance of a balanced pruning approach, one that minimizes model size while maintaining or enhancing performance. Considering the impact of different pruning thresholds on the model, the thresholds T for all other layers are uniformly set to 0.05.

In conclusion, the findings emphasize the need for careful tuning of the pruning threshold to achieve the right balance between efficiency and accuracy. This balance is especially crucial in applications where both computational resources and model performance are paramount, such as in mobile or edge devices.

#### 4.3.2. Impact of Different Regularization

To evaluate the effectiveness of the proposed hybrid regularization method, we conducted an ablation study comparing its performance to that of $L_{1}$ and $L_{2}$ regularization. The experiments were carried out on the VGG-16 model using the CIFAR-10 dataset, with a pruning threshold T of 0.05 and a regularization strength λ of $10^{-5}$ for all methods, as shown in Table 7. The results revealed that $L_{1}$ regularization achieved a classification accuracy of 93.69%, while $L_{2}$ regularization yielded a marginally higher accuracy of 93.79%. In contrast, the hybrid regularization method, which combines the advantages of both $L_{1}$ and $L_{2}$ regularization, achieved the highest accuracy of 93.93%. This demonstrates that the hybrid regularization approach provides a consistent, albeit modest, improvement in classification performance over the traditional $L_{1}$ and $L_{2}$ methods. Overall, the findings suggest that the hybrid regularization method strikes a better balance between sparsity and feature retention, leading to enhanced model efficiency and generalization without compromising performance.

#### 4.3.3. Impact of Information Extraction Module

To evaluate the effectiveness of the proposed information extraction module (IEM), we conducted an ablation study comparing the compression results and performance of models with and without the IEM module. The experiments were carried out on the CIFAR-10 dataset, using both the VGG-16 and ResNet-56 models. A pruning threshold T of 0.05 and a regularization strength λ of $10^{-5}$ were used in the experiments.

As shown in Table 8, the inclusion of the IEM module leads to a reduction in both the number of parameters and computational complexity while maintaining the model’s accuracy. Specifically, for the VGG-16 model, the IEM module achieves the same accuracy as the baseline model but reduces the number of parameters from 1.28 M to 0.81 M and decreases FLOPs from 106.82 M to 75.73 M, demonstrating a notable improvement in compression efficiency.

Similarly, for the ResNet-56 model, the IEM module slightly improves accuracy from 93.61% to 93.65%, while reducing the number of parameters from 0.39 M to 0.35 M, and achieving a minor reduction in FLOPs from 49.5 M to 49.2 M. These results highlight the ability of the IEM module to effectively maintain or even enhance model performance, while significantly improving the compression rate, particularly in terms of parameter reduction.

Overall, the inclusion of the IEM module demonstrates its capacity to balance information flow within the network, making it a valuable component for improving both the compression performance and efficiency of deep neural networks. The results underscore its effectiveness in enhancing model sparsity without compromising performance.

### 4.4. Network Visualization

Deep neural networks (DNNs) have achieved revolutionary success in computer vision, demonstrating outstanding performance across a wide range of tasks. However, their “black-box” nature presents significant challenges for both research and practical deployment. To address these challenges, visualization techniques have become essential tools, enabling detailed inspection of the network’s internal workings, performance evaluation, and a deeper understanding of the features learned during training.

**Figure 4:**
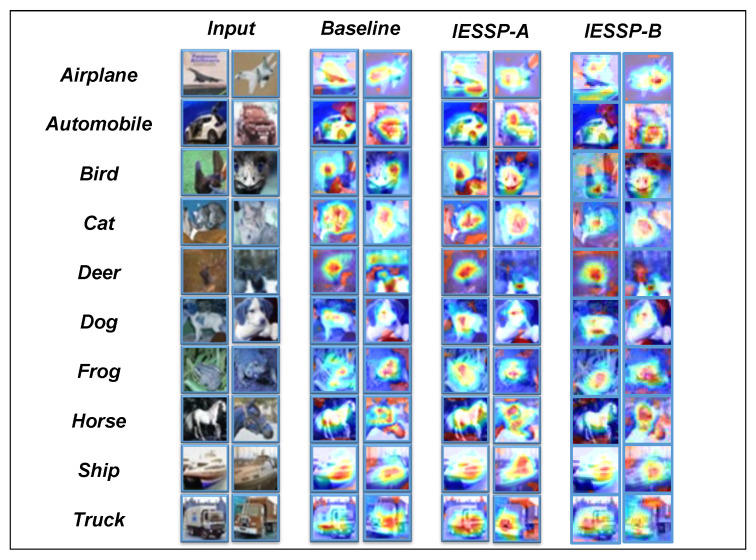
Several CIFAR-10 test images and the Grad-CAM results on ResNet-20.

**Figure 5:**
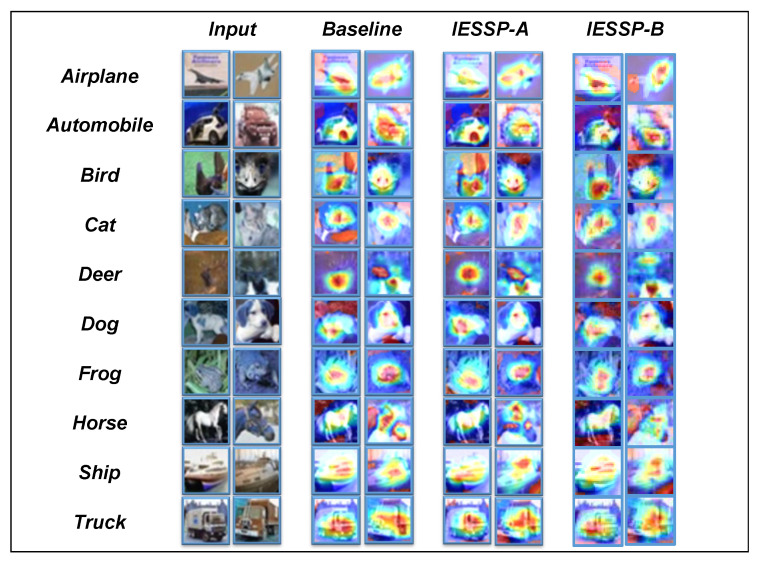
Several CIFAR-10 test images and the Grad-CAM results on ResNet-32.

**Figure 6:**
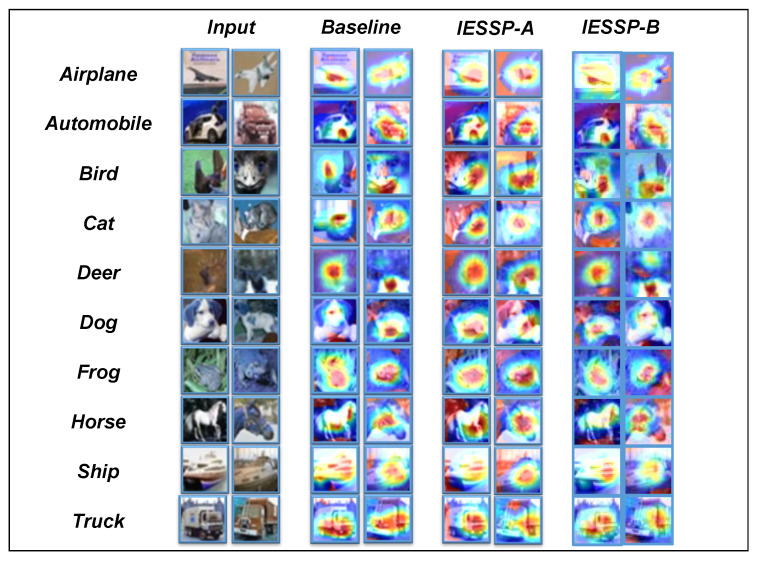
Several CIFAR-10 test images and the Grad-CAM results on ResNet-56.

In this study, we introduce Class Activation Maps (CAMs) [60] as the primary visualization method for analyzing pruned networks. A CAM is an effective technique for exploring the decision-making process of networks in image classification tasks. By highlighting the regions of the input image that most significantly influence the network’s predictions, CAM provides an in-depth analysis. As demonstrated in Figure 4, Figure 5 and Figure 6, the CAM visualizations on the CIFAR-10 dataset show that the pruned network focuses on the most relevant regions for classification.

Although this section is primarily qualitative, our experimental observations reveal that for instances within the same category, the network consistently activates similar regions of the image. Specifically, we observed that samples from different models concentrate on similar regions of the image, indicating that, despite the pruning process, the network retains its ability to extract important features effectively.

In conclusion, the use of CAM not only validates the effectiveness of the proposed pruning method but also provides valuable insights into the internal workings of the network, contributing to a deeper understanding of neural network operations and advancing future developments in network design and performance optimization.

## 5. Conclusions

This paper presents a novel information extraction sparse stripe pruning method (IESSP) that effectively addresses the challenges of over-regularization and inaccurate stripe selection in network pruning. By incorporating an information extraction module during sparse training, IESSP adaptively adjusts sparsity levels, aligning sparsity control with the primary training objective for a more efficient pruning process. Additionally, the method utilizes network-level information to identify model redundancies more effectively, resulting in improved feature selection compared to conventional pruning techniques. Experimental results on the CIFAR-10 and ILSVRC-2012 datasets demonstrate that IESSP achieves improvements in both model compression performance and computational efficiency. For instance, in classification experiments using ResNet-18 on the ILSVRC-2012 dataset, our method achieves a 48.25% reduction in model size and a 56.59% increase in model speedup while maintaining a minimal accuracy drop of only 1.09% compared to the baseline model. IESSP shows significant promise for deployment in mobile devices, embedded systems, and edge computing. Future research will focus on combining IESSP with other techniques like quantization to further improve model compression and performance. We also plan to explore using IESSP with other acceleration methods to optimize computational resources and enhance deep neural network performance.

## Figures and Tables

**Table 1:** A comprehensive parameter table for the baseline dataset and its corresponding training model is provided.

Structure	Dateset	Pruned Layer	Weights	FLOPs	Topology	Output Candidates
VGG-16 [48]	CIFAR-10 [47]	16	15 M	314 M	13CONV + 3FC	10
ResNet-20 [49]	CIFAR-10 [47]	20	0.27 M	41.1 M	19CONV + 1FC	10
ResNet-32 [49]	CIFAR-10 [47]	32	0.46 M	69.8 M	31CONV + 1FC	10
ResNet-56 [49]	CIFAR-10 [47]	56	0.85 M	127 M	55CONV + 1FC	10
ResNet-18 [49]	ILSVRC-2012 [50]	18	11.69 M	1.82 B	17CONV + 1FC	1000
ResNet-34 [49]	ILSVRC-2012 [50]	34	21.8 M	3.68 B	33CONV + 1FC	1000

**Table 2:** Various network architectures were employed to demonstrate the performance of the proposed method on the CIFAR-10 dataset. “Acc ↑↓” represents the change in accuracy, where an upward arrow indicates an increase and a downward arrow indicates a decrease. “Pruned ↓” denotes the compression ratio, defined as the ratio of remaining weights to the original number of weights. The “Speedup” ratio is defined as the ratio of the trimmed FLOPs to the original FLOPs. A hyphen (“-”) indicates that no results are available.

Model	Acc	Acc ↑↓	FLOPs	Speedup	Parameters	Pruned ↓
VGG-16 Baseline [48]	93.40%	-	314 M	-	15 M	-
IESSP	**93.69%**	**0.29↑ %**	**75.73 M**	**75.88%**	**0.81 M**	**94.6%**
ResNet-20 Baseline [49]	**92.05%**	-	41.1 M	-	0.27 M	-
IESSP	91.27%	0.78↓ %	**16.0 M**	**60.6%**	**0.13 M**	**51.9%**
ResNet-32 Baseline [49]	92.82%	-	69.8 M	-	0.46M	-
IESSP	**92.84%**	**0.02↑ %**	**27.6 M**	**60.5%**	**0.21 M**	**54.3%**
ResNet-56 Baseline [49]	93.06%	-	127 M	-	0.85 M	-
IESSP	**93.65%**	**0.59↑ %**	**49.2 M**	**61.3%**	**0.35 M**	**58.8%**

**Table 3:** Multiple network architectures were employed to showcase the performance of te proposed method on the ILSVRC-2012 dataset. “Acc ↑↓” represents the change in accuracy, where an upward arrow indicates improvement and a downward arrow indicates degradation. “Pruned ↓” refers to the compression ratio. “Acc-1” and “Acc-5” denote the top 1 and top 5 accuracies, respectively. A hyphen (“-”) indicates that no results are available.

Model	Acc-1	Acc-1 ↑↓	Acc-5	Acc-5 ↑↓	FLOPs	Speedup	Parameters	Pruned ↓
ResNet-18 Baseline [49]	**70.42%**	-	**89.63%**	-	1.82 B	-	11.69 M	-
IESSP	69.33%	1.09↓ %	89.07%	0.56↓ %	**6.05 B**	**56.59%**	**6.05 M**	**48.25%**
ResNet-34 Baseline [49]	**74.01%**	-	**91.65%**	-	3.68 B	-	21.80 M	-
IESSP	73.18%	0.83↓ %	91.26%	0.39↓ %	**1.51 B**	**58.96%**	**10.75 M**	**53.90%**

**Table 4:** Comparison of IESSP with state-of-the-art model compression methods on ILSVRC-2012. “Acc ↑↓” indicates the change in accuracy, where an upward arrow denotes improvement and a downward arrow denotes reduction. “Pruned ↓” represents the compression ratio. “Acc-1” and “Acc-5” correspond to the top 1 and top 5 accuracies, respectively. “-” indicates that no results were reported. A_Acc represents the average accuracy of the model. The highest value in each column is shown in bold, and the second-highest value is underlined.

Network	Method	Param	FLOPs	Acc	Parameters ↓	FLOPs ↓	Acc ↑	A_Acc
VGG-16	Baseline [48]	15.0 M	314 M	93.40%	-	-	-	93.64%
	PFEC [24]	5.4 M	206 M	93.40%	64%	34.39%	0.00%	
	FPGM [30]	5.4 M	206 M	93.54%	64%	34.39%	0.14%	
	GAL [52]	3.36 M	189 M	93.77%	77.6%	39.81%	0.37%	
	HRank [31]	2.51 M	146 M	93.43%	83.3%	53.50%	0.03%	
	SWP [43]	1.10 M	90.6 M	93.65%	92.6%	71.15%	0.25%	
	ASK_3a [44]	5.18 M	106 M	93.89%	64.46%	66.24%	0.49%	
	SOKS-80% [45]	3.25 M	87.3 M	**94.01%**	78.3%	72.20%	**0.61%**	
	EACP [53]	3.42 M	**71 M**	93.20%	77.2%	**77.40%**	−0.20%	
	SCP [54]	1.36 M	99.5 M	93.87%	90.9%	68.31%	0.47%	
	IESSP	**0.81 M**	75.73 M	93.69%	**94.6%**	75.88%	0.29%	
ResNet-20	Baseline [49]	0.27 M	41.1 M	92.05%	-	-	-	91.20%
	MIL [55]	-	32 M	91.43%	-	22.14%	−0.62%	
	SFP [19]	-	24.3	90.83%	-	40.88%	−1.22%	
	FPGM [30]	-	24.3	91.09%	-	40.88%	−0.96%	
	ASK_5b [44]	0.15 M	23.1 M	**91.78%**	44.4%	43.80%	**−0.27%**	
	SOKS-80% [45]	0.14 M	**15.6 M**	90.78%	48.14%	**62.40%**	−1.27%	
	IESSP	**0.13 M**	16.0 M	91.27%	**51.85%**	61.07%	−0.78%	
ResNet-32	Baseline [49]	0.46 M	69.8 M	92.82%	-	-	-	91.76%
	MIL [55]	-	47 M	90.74%	-	32.66%	−2.08%	
	SFP [19]	-	40.3 M	90.08%	-	42.26%	−2.74%	
	FPGM [30]	-	40.3 M	92.31%	-	42.26%	−0.51%	
	ASK_5b [44]	0.26 M	23.1 M	92.19%	43.48%	66.91%	−0.63%	
	SOKS-80% [45]	0.21 M	31.7 M	92.02%	54.35%	54.58%	−0.80%	
	SCP [54]	**0.16 M**	**17.38 M**	92.17%	**65.22%**	**75.10%**	−0.65%	
	IESSP	0.21 M	27.6 M	**92.84%**	54.35%	60.46%	**0.02%**	
ResNet-56	Baseline [49]	0.85 M	127 M	93.06%	-	-	-	92.98%
	PFEC [24]	0.73 M	90.9 M	93.06%	14.12%	28.43%	0.00%	
	NISP [51]	0.49 M	71.6 M	92.99%	42.35%	43.62%	−0.07%	
	GAL [52]	0.29 M	50 M	91.58%	65.88%	60.63%	−1.48%	
	HRank [31]	0.49 M	62.7 M	93.17%	42.35%	64.30%	0.11%	
	SWP [43]	**0.19 M**	**31.0 M**	92.98%	**77.64%**	**75.59%**	−0.08%	
	ASK_3a [44]	0.48 M	71.3 M	93.02%	43.53%	43.86%	−0.04%	
	SOKS-80% [45]	0.39 M	61.3 M	93.08%	54.18%	51.73%	0.02%	
	EACP [53]	0.31 M	40.6 M	93.11%	63.53%	68.03%	0.05%	
	SCP [54]	0.34 M	40.5 M	93.11%	60.00%	68.11%	0.05%	
	IESSP	0.35 M	49.2 M	**93.65%**	58.82%	61.26%	**0.59%**	

**Table 5:** Comparison of IESSP with state-of-the-art model compression methods on ILSVRC-2012. “Acc ↑↓” indicates the change in accuracy, where an upward arrow denotes improvement and a downward arrow denotes reduction. “Pruned ↓” represents the compression ratio. “Acc-1” and “Acc-5” correspond to the top 1 and top 5 accuracies, respectively. “-” indicates that no results were reported. A_Acc1 represents the average top 1 accuracy of the model. The highest value in each column is shown in bold, and the second-highest value is underlined.

Network	Method	Parameters	FLOPs	Acc-1	Acc-5	Parameters ↓	FLOPs ↓	Acc-1 ↓	Acc-5 ↓	A_Acc1
ResNet-18	Bseline [49]	11.69 M	1.82 B	70.42%	89.63%	-	-	-	-	68.51%
	MIL [55]	-	1.19 B	66.33%	86.94%	-	34.6%	4.09%	2.69%	
	SFP [19]	-	1.06 B	67.10%	87.78%	-	41.8%	3.32%	1.85%	
	FPGM [30]	-	1.06 B	68.34%	88.53%	-	41.8%	3.32%	1.10%	
	COP [56]	6.42 M	1.03 B	66.98%	-	45.1%	43.3%	3.44%	-	
	DMCP [57]	-	1.04 B	69.2%	-	-	42.9%	1.22%	-	
	TAS [58]	-	1.21 B	69.15%	89.19%	-	33.52%	1.27%	0.44%	
	SWP [43]	-	0.90 B	**69.99%**	89.30%	-	50.48%	**0.43%**	**0.33%**	
	ASK_3a [44]	6.81 M	1.08 B	69.55%	88.98%	41.75%	40.66%	0.87%	0.65%	
	SOKS [45]	6.27 M	0.82 B	69.16%	88.81%	46.36%	54.95%	1.26%	0.82%	
	IESSP	**6.05 M**	**0.79 B**	69.33%	89.07%	**48.25%**	**56.59%**	1.09%	0.56%	
ResNet-34	Bseline [49]	21.80 M	3.68 B	74.01%	91.65%	-	-	-	-	72.71%
	PFEC [24]	19.3 M	2.76 B	72.17%	-	11.5%	25.0%	1.10%	-	
	Rethinking [59]	19.3 M	2.76 B	72.91%	-	11.5%	25.0%	1.10%	-	
	MIL [55]	-	2.77 B	72.99%	91.19%	-	24.8%	1.02%	0.46%	
	SFP [19]	-	2.17 B	71.83%	90.33%	-	41.1%	2.18%	1.32%	
	FPGM [30]	-	2.17 B	72.54%	91.13%	-	41.1%	1.47%	0.52%	
	NISP [51]	12.28 M	2.07 B	71.65%	-	43.68%	43.76%	2.36%	-	
	Taylor [28]	17.2 M	2.83 B	72.83%	-	21.10%	23.10%	1.18%	-	
	ASK_3a [44]	12.43 M	2.11 B	73.44%	**91.47%**	42.98%	42.66%	0.57%	**0.18%**	
	SOKS [45]	11.27 M	1.62 B	**73.52%**	91.35%	48.30%	55.98%	**0.49%**	0.30%	
	IESSP	**10.75 M**	**1.51 B**	73.18%	91.26%	**53.90%**	**58.96%**	0.83%	0.39%	

**Table 6:** Comparison of the effects of different threshold T [43] to the VGG-16 model on CIFAR-10.

T	Param	FLOPs	Acc
0.03	3.24 M	159 M	94.16%
0.04	2.42 M	147 M	93.92%
0.05	1.31 M	107 M	93.82%
0.06	1.01 M	93 M	93.75%

**Table 7:** Comparison of the effects of different regularization to the VGG-16 model on CIFAR-10. A “✓” denotes the inclusion of the corresponding regularization, while a “✗” indicates its exclusion.

$L_{ce}$	$L_{1}$	$L_{2}$	$L_{S}$	Acc
✓	✗	✗	✗	93.40%
✓	✓	✗	✗	93.69%
✓	✗	✓	✗	93.79%
✓	✗	✗	✓	93.93%

**Table 8:** Comparison of the effects of information extraction module to the VGG-16 and ResNet-56 model on CIFAR-10. The “✓” indicates the use of the information extraction module, while the “✗” indicates the absence.

Model	IEM	Acc	FLOPs	Parameters
VGG-16	✗	93.69%	106.82 M	1.28 M
VGG-16	✓	93.69%	75.73 M	0.81 M
ResNet-56	✗	93.61%	49.5 M	0.39 M
ResNet-56	✓	93.65%	49.2 M	0.35 M

## Data Availability

All datasets used are available online with open access.
